# On Cataract, and Its Appropriate Treatment by the Operation Adapted to Each Case

**Published:** 1845-10

**Authors:** 


### Art. XXI.

*On Cataract, and its appropriate Treatment by the Operation adapted to each Case.* By Charles Gardiner Guthrie, Assistant-Surgeon to the Royal Westminster Ophthalmic Hospital.—*London,* 1845. 8vo, pp. 128.

We have redd this book with surprise and regret,—we might indeed say, with astonishment and sorrow. If, on being made acquainted with the circumstances which have excited these feelings, the profession should decide that they are reasonable and just, then, assuredly, has the ostensible author of this volume incurred a heavy responsibility towards the order to which he belongs, which it behoves him to lose no time in attempting to discharge. It will afford us very great pleasure to find that he is able to effect what is required of him, in a way that can be satisfactory to his brethren and himself. He may rest assured that this is no light matter; his own honour as well as the dignity of the medical profession alike demand vindication at his hands.

The title-page of Mr. Charles Guthrie's book we have transcribed above. The following is an equally correct transcript of the preface:

"The following observations contain the substance of that part of the Lectures on Surgery which have been for years delivered by Mr. Guthrie to the Medical Officers of the Public Service, and to the Students of the Westminster and the

Royal Westminster Ophthalmic Hospitals; as well as of those Clinical Lectures given by me at the Hospital during the last winter, which relate to the treatment of Cataract. I have endeavoured to be explicit in enumerating the symptoms and appearances of each kind, and in the directions explanatory of the different operations most appropriate for them; with the hope that they cannot be mistaken by professional readers, whilst they are as far as possible divested of unnecessary technicalities, that they may be readily understood by every intelligent person desirous of becoming acquainted with this interesting subject.

"19, *Savile Row;* May 20, 1845. Charles Gardiner Guthrie."

Now, from this title-page and this preface, have we not a right to expect that the work to which they are prefixed, is the work of Mr. Charles Guthrie? Would it ever occur to any reader to think otherwise? The utmost that any one ignorant of the fact could expect from the statement, would be—that the author had availed himself of the *unpublished* lectures of Mr. Guthrie, senior, and only of the *substance* of these. He never would imagine, from the wording of this preface, that these lectures, which "have been for years delivered by Mr. Guthrie," had ever previously, even in *substance,* been communicated to the members of the profession generally. He would also, of course, conclude that the *substance* of "the clinical lectures given at the hospital during the last winter" by Mr. Charles Guthrie, would truly have some *substance* of their own, to justify their being placed in the same category and on the same level as those "delivered to the medical officers of the public service,"—by his father. Finally, seeing only in the expressions "*I have endeavoured* to be explicit," &c. . . . "*with the hope,*" &c. the simple and natural avowal of the responsibility attaching to original authorship, he would necessarily infer that whatever assistance Mr. Charles Guthrie might derive from the facts and doctrines supplied by others, the working-up of the materials was at least his own act, and, *a fortiori,* the statements, opinions, judgments, directions, reflections contained in the book, and enunciated, as it were, through his own lips, by his own pen, and authenticated by the unequivocal evidence of the personal pronoun *I,*—were, at least, his own statements, opinions, judgments, directions, reflections. To imagine otherwise, would seem not merely absurd, but would be at once felt to be incredible—yea impossible!

And yet what is the fact? Charles Gardiner Guthrie is just as much the author of this book as the Editor of this Journal is the author of all the articles contained in its Forty Numbers! Nay, not so much; for there is no single article—no single page, we were going to say, certainly no three consecutive pages in the book,—nay not ten pages of the whole hundred and twenty-seven, written at all by him, its ostensible author! This is most strange—yet it is true: "Truth is stranger than fiction."

Most of our older readers are aware that Mr. G. J. Guthrie, the father of Mr. Charles Guthrie, published an excellent work on diseases of the eye, in the year 1823, entitled 'Lectures on the Operative Surgery of the Eye; or an historical and critical inquiry into the methods recommended for the cure of Cataract,' &c. It was reprinted in 1827; and this, the second edition, is the one now before us. In the year 1836 Mr. Guthrie also published a small pamphlet of fifty pages, 'On the Certainty and Safety with which the operation for extraction of a Cataract from the Human Eye may be performed, and on the means by which it is to be accomplished.' It is out of these two BOOKS—books in the hands of all old surgeons,—and out of no private and peculiar store of paternal manuscripts, that Mr. Charles Guthrie has framed his volume! The preface tells us nothing of the pamphlet, though the *substance* of this is also "contained" in it. And well and truly may Mr. Charles Guthrie assure us that his book "contains," the substance of his father's "Lectures,"—since it is framed from them, precisely on the same plan and pattern as young Paddy frames his jacket by hacking off all superfluity of tail and sleeve from the paternal long-coat; or as his honest sire frames his pavement in Piccadilly or Pall Mall, by restoring to their resting-place anew, the very identical blocks which he had displaced the day before. Like Paddy's blocks of granite, the contents of Mr. Guthrie's books are transferred, *en masse,* to the pages of his son's. Sometimes, indeed, they may receive a little chipping, here and there, to make them fit; or a little original rubbish may be thrown in to fill up unseemly vacancies; and a piece may be occasionally turned topsy-turvy or athwart, or shifted from its original locality; still all must admit, that everything has been handled with the most filial reverence, and that the author and the paviour have been alike successful in achieving that most difficult of mortal tasks, a real practical bull—*a thing another yet the same.*

Of Mr. Charles Guthrie's volume the first sixty pages are taken from the 'Operative Surgery;' the next twenty-four from the 'Certainty and Safety' pamphlet; and the rest of the volume from p. 84 to *Finis,* again from the 'Operative Surgery:'—always allowance being made for the chippings and the rubbish aforesaid.

Were the subject not, in reality, a very serious and melancholy one, a comical exhibition might be made by placing in juxta-position all the statements, and directions, and reflections of Mr. Guthrie and Mr. Charles Guthrie. In contemplating them together, sober common-places become irresistibly ludicrous from mere identity; unanimity of sentiment makes one laugh by its very perfection.

"The pressure of the forefinger tends to fix the eye at the same time, and to render it as immoveable as possible, and this mode of proceeding," *says* Mr. Guthrie, "I generally adopt in preference for the left eye." (*On the Certainty, &c.* p. 10.)

"The pressure of the forefinger tends, &c. .... and this mode of proceeding," *says* Mr. Charles Guthrie, "*I* generally adopt in preference for the left eye." (*On Cataract,* p. 64.)

"Where persons are naturally irritable or nervous," *says* Mr. Guthrie, "I always separate the lids, and fix the eye two or three times at an interval of two or three days." (*On the Certainty, &c.* p. 14.)

"Where persons are naturally irritable or nervous," *says* Mr. Charles Guthrie, *I* always separate the lids," &c. (*On Cataract,* p. 66.)

"This is the acme of perfection in operating," *says* Mr. Guthrie; "I am aware that it cannot always be done." (*On the Certainty, &c.* p. 20.)

"This is the acme of perfection in operating," *says* Mr. Charles Guthrie; "*I* am aware it cannot always be done." (*On Cataract,* p. 70.)

"In very nervous or agitated persons," *says* Mr. Guthrie, "I always leave," &c. (*On the Certainty, &c.* p. 26.)

"In very nervous or agitated persons," *says* Mr. Charles Guthrie, "*I* always leave," &c. (*On Cataract*, p. 74.)

"I have seen cases," *says* Mr. Guthrie, "in which little or scarcely any deviation from the proper situation of the pupil has taken place," &c. (*On the Certainty, &c.* p. 35.)

"*I* have seen cases," *says* Mr. Charles Guthrie, "in which little or scarcely any deviation from the proper situation of the pupil has taken place," &c. (*On Cataract*, p. 78.)

"From a due consideration of these two circumstances, with the almost impossibility of avoiding them both, I have no hesitation," *says* Mr. Guthrie, "in condemning this method," &c. (*Operative Surgery*, p. 294.)

"From a due consideration of these two circumstances, with the almost impossibility of avoiding them both, *I* have no hesitation," *says* Mr. Charles Guthrie, "in condemning this method," &c. (*On Cataract*, p. 89.)

"New feelings will arise," piously exclaims Mr. Guthrie, "in admiration of the benignity of the Creator," &c. (*On the Certainty, &c.* p. 66.)

"New feelings will arise," piously exclaims Mr. Charles Guthrie; "in admiration of the benignity of the Creator," &c. (*On Cataract*, p. 13.)

"But he is not the person in error," *says* Mr. Guthrie; "it is those who wrote the directions. I do not mean to say," *adds* Mr. Guthrie, "*that these gentlemen have willingly deceived the public*, but I do say it is my opinion, that they have told only half the truth." (*On the Certainty, &c.* p. 22.)

"He is not, however, the person in error," *says* Mr. Charles Guthrie; "it is those who wrote the directions. *I* do not mean," *adds* Mr. Charles Guthrie, "*that these gentlemen have willingly deceived the public*, but it is *my* opinion they have told only half the truth." (*On Cataract*, p. 72.)

And so Mr. Guthrie and Mr. Charles Guthrie proceed from beginning to end,—identical in word, opinion, judgment, sentiment; exhibiting such an instance of paternal and filial union and unanimity as, we will venture to say, was never before presented, in history or fiction, in prose or rhyme, since the world began.

Nor is this identification even confined to words; the very pictures are the same. In pages 22-24 of Mr. Charles Guthrie's book, we have three diagrams, and a letter from Capt. Kater. In Mr. Guthrie's pamphlet, (pp. 45-7,) we have the same letter and the same three diagrams! To be sure, Mr. Charles Guthrie has turned the diagrams topsy-turvy; and he gives the observations as Capt. Kater's, and as "addressed to Mr. Guthrie in the year 1834;" but he leaves the reader to imagine (if he pleases,) how this letter "of the late Capt. Kater" and its woodcuts, got into his hands; not a word is said of the pamphlet, and nobody but such old readers as ourselves would ever dream of the letter having been published before. And we may as well remark here, that from the beginning of Mr. Charles Guthrie's book to the end, his father's writings are never once quoted, never once referred to! Every now and then, indeed, "Mr. Guthrie" is mentioned by name as having seen or done so and so, when the employment of the personal pronoun of the original, would "be coming it rather too strong," even for Mr. Charles Guthrie. For example, "Mr. Guthrie"

says Mr. Charles Guthrie, (p. 61,) "took some pains to correct this little irregularity. . . . This trifle constitutes one of the greatest improvements, &c." "I have taken some pains" says Mr. Guthrie, (Pamphlet, p. 29,) to correct this little irregularity. . . . . . This trifle constitutes one of the greatest improvements," &c.

Again, at the end of Mr. Charles Guthrie's book, there is a grand display of staring eyes, nine in number, in a copper-plate, with a page of explanation, and references to the pages of his book the statements in which they are intended to illustrate. On turning to Mr. Guthrie's 'Operative Surgery,' we were stared into a state of almost mesmeric fascination, by the same nine horrid eyes, each in the same identical spot as in Mr. Charles's book, and all goggling at us with the same genuine ophthalmological ghastliness! And is the plate really the same? Oh no! not the same; but it is one more brilliant example of the practical bull—*it is another yet the same.* The nine eyes to be sure are there; the place, the shape, the size, the number of each—all are identical;—but, then—ay then—*the colour of the irides is different*—and *the imprint,*—"Engraved by I. Stewart,—Published May 1827, by Burgess and Hill," *is not there.* How then can it be the same plate?

But we must resume the tone that best becomes the subject, and here close our painful task;—a task which nothing but the strongest sense of duty, imposed upon us by the responsibilities of the position which we occupy, could have ever led us to undertake. It seems probable that such a thing as we have been called upon to expose, was never done before; we hope, for the sake of our profession, it will never be done again in medical literature; we cannot imagine why it has been done now; and, although we see that it is done, we know neither the history nor the mystery of its doing. One thing is clear, however, that the profession will expect some further explanation of the circumstance; and that this explanation must extend to both the real and ostensible author of the volume before us. Either the two Messrs. Guthrie, father and son, were mutually cognisant and participant of the fact and act; or the son has been injuring the father without his knowledge. If Mr. Guthrie, senior, was a knowing and a consenting party, on what principle, we ask, can he justify the re-issue of a portion of his own writings under the name of another, though that other be his son? Or on what principle can Mr. Charles Guthrie, even with his father's consent, avow himself the author of the works of another, though that other be his father? On the other hand, if all has been done, without Mr. Guthrie's knowledge, then we must regard Mr. Charles Guthrie as doubly responsible—in thus deepening, as he does, the sin of a most monstrous plagiarism, by committing the robbery on one whose literary property and fame he, above all others, was bound to respect and defend.

